# A non-canonical monovalent zinc finger stabilizes the integration of Cfp1 into the H3K4 methyltransferase complex COMPASS

**DOI:** 10.1093/nar/gkz1037

**Published:** 2019-11-14

**Authors:** Yidai Yang, Monika Joshi, Yoh-hei Takahashi, Zhibin Ning, Qianhui Qu, Joseph S Brunzelle, Georgios Skiniotis, Daniel Figeys, Ali Shilatifard, Jean-François Couture

**Affiliations:** 1 Shanghai Institute of Materia Medica-University of Ottawa Joint Research Centre on Systems and Personalized Pharmacology, University of Ottawa, Ottawa, ON K1H 8M5, Canada; 2 Ottawa Institute of Systems Biology and Department of Biochemistry, Microbiology and Immunology , University of Ottawa, Ottawa , ON K1H 8M5 , Canada; 3 Department of Biochemistry and Molecular Genetics, Northwestern University, Chicago, IL 60611, USA; 4 Departments of Molecular and Cellular Physiology, and Structural Biology, Stanford University School of Medicine, Stanford, CA 94305, USA; 5 Northwestern Synchrotron Research Centers, Life Science Collaborative Access Team, Northwestern University, Evanston, IL, USA

## Abstract

COMPlex ASsociating with SET1 (COMPASS) is a histone H3 Lys-4 methyltransferase that typically marks the promoter region of actively transcribed genes. COMPASS is a multi-subunit complex in which the catalytic unit, SET1, is required for H3K4 methylation. An important subunit known to regulate SET1 methyltransferase activity is the CxxC zinc finger protein 1 (Cfp1). Cfp1 binds to COMPASS and is critical to maintain high level of H3K4me3 in cells but the mechanisms underlying its stimulatory activity is poorly understood. In this study, we show that Cfp1 only modestly activates COMPASS methyltransferase activity *in vitro*. Binding of Cfp1 to COMPASS is in part mediated by a new type of monovalent zinc finger (ZnF). This ZnF interacts with the COMPASS’s subunits RbBP5 and disruption of this interaction blunts its methyltransferase activity in cells and *in vivo*. Collectively, our studies reveal that a novel form of ZnF on Cfp1 enables its integration into COMPASS and contributes to epigenetic signaling.

## INTRODUCTION

Histone H3 K4 methylation (H3K4) is a post-translational modification (PTM) deposited by an evolutionary conserved family of lysine methyltransferases referred to as the lysine Methyl-Transferase 2 (KMT2) (1). Also referred to as COMPlex Associated to SET1 (COMPASS), six human KMT2 enzymes contribute, to different extent, to the deposition of this PTM. While each member is linked to different biological functions (1), the methyltransferase activity of each member is exquisitely dependent on an obligate association to a four-subunit complex composed of Ash2L (also referred to as Bre2 or Cps60), RbBP5 (also referred to as Swd1 or Cps50), WDR5 (also referred to as Swd3 or Cps30) and DPY-30 (also referred to as Sdc1 or Cps25) (2–5). In addition, each KMT2 enzyme specifically associates to unique subunits. For example, Menin binds the N-terminus of MLL1 to regulate *hox* gene expression in MEF cells (6). Binding of NCOA6 to MLL3 and MLL4 regulates adipogenesis (7,8) and interaction of WDR82 (also referred to as Swd2 or Cps35) with SET1A/B mediates its association to RNA polymerase (9). Finally, the ability of Cfp1 to associate with SET1A/B (10) and unmethylated CpG islands increases the retention of COMPASS to chromatin (11).

Cfp1 controls the development of mouse oocyte (12) and meiotic cell cycle progression (13). Hematopoietic stem cells depleted in Cfp1 fail to undergo hematopoietic differentiation leading to a severe loss of commitment to a specific lineage and formation of mature cells (14). Cfp1 also interacts with IHO1, an essential component of the meiotic double-strand break (DSB) machinery (15) and its yeast homolog (also referred to as Spp1 or Cps40), associates with Mer2 (16) to participate in DNA damage repair (16,17). Cfp1 helps the recruitment of SET1 enzymes to unmodified CpG dinucleotide (18,19). Moreover, yeast cells lacking Cps40 loose 80% of global H3K4 trimethylation without significant changes on H3K4 mono- and di-methylation (20). Detailed genome wide studies fine-tuned this model in showing that the CpG islands (CGIs) expressed genes are affected by the loss of Cfp1 and that its DNA binding activity prevents random deposition of H3K4me3 at regulatory regions (21).

Mechanistically, an evolutionary conserved motif, referred to as SET interacting domain (SID), of Cfp1 directly associates with the N-terminus of SET1 (residues 762–937 in yeast) (22,23) and helps maintaining Cfp1 bound to SET1 complexes in the nucleus (11). In vertebrates, Cfp1 CXXC1 domain binds unmethylated DNA (24) and a PHD domain located on its N-terminus binds H3K4me3 (11,25). A recent cryo-EM structure of COMPASS (26) places Cfp1 in close proximity to RbBP5, WDR5 and the N-terminus of SET1 but the determinants underlying its integration in the complex are unclear. Here we set out to identify the minimal structural determinants underlying the integration of Cfp1 into COMPASS. Structural, biochemical and *in vivo* studies show that a novel zinc finger (ZnF) on Cfp1 interacts with an evolutionary conserved pocket located on the edge of RbBP5 β-propeller domain. Mutation of key residues forming the interface disrupts the integration of Cfp1 into COMPASS complexes and disrupts H3K4me3 in mammalian and yeast cells. Moreover, structural analysis reveals that this ZnF, which is found in the region that interacts with RbBP5 (also referred to as RbBP5 Interacting Domain (RID), adopts a novel topology that does not resemble the PHD domain observed in other histone binding proteins nor any hitherto characterized ZnF.

## MATERIALS AND METHODS

### Protein expression, purification and CtCOMPASS reconstitution

Full-length (FL) *Chaetomium thermophilum* (Ct) Cfp1, WDR5, Ash2L and *Myceliophteria thermophila* (Mt) RbBP5 and its β-propeller (residues 1–347) were cloned into pET28. A CtSET1 fragment containing its nSET and SET domains (residues 966–1295) was cloned into pSMT3 while full-length or a fragment corresponding to the RID domain of CtCfp1 (residues 322–508) were cloned into pGEX. All these proteins were overexpressed in *Escherichia coli* Rosetta cells (Novagen) using 0.2 mM isopropylthiogalactopyranoside (IPTG) during 16 h at 18°C. Following overexpression, cells were lysed by sonication in a lysis buffer containing 50 mM sodium phosphate pH 7.0, 500 mM sodium chloride and 5 mM β-mercaptoethanol. Cells expressing CtWDR5 were lysed in 20 mM HEPES pH 7.0, 500 mM sodium chloride, 5 mM β-mercaptoethanol, and 1% Triton. Proteins were purified by affinity chromatography in lysis buffer and eluted with 50 mM sodium phosphate pH 7.0, 500 mM sodium chloride, 5 mM β-mercaptoethanol and 500mM imidazole. Due to poor stability and solubility of untagged CtWDR5, the protein was mixed with MtRbBP5 in a 1.5:1 molar ratio prior TEV cleavage. Following the removal of the hexahistidine tag, the complex was further purified by size exclusion chromatography (SEC) using a Superdex 200 (GE Healthcare) pre-equilibrated in a buffer containing 50 mM Tris pH 8.0, 200 mM sodium chloride and 5 mM β-mercaptoethanol. Similarly, purified CtSET1 and CtAsh2L were mixed in a 1:1 molar ratio prior cleavage and co-purified using a Superdex 200. CtCfp1 and its RID domain were purified as previously described (26). The CtWDR5/MtRbBP5/CtSET1/CtAsh2L complex was reconstituted by mixing the two heterodimers in a 1:1 molar ratio, incubated at 4°C during 4 h and purified by SEC using a Superdex 200 pre-equilibrated in 50 mM Tris pH 8.0, 150 mM sodium chloride and 5 mM β-mercaptoethanol. The CtWDR5/CtAsh2L/MtRbBP5/CtSET1 complex was then mixed with CtCfp1 in a 1:5 molar ratio, incubated at 4°C during 2 h and further purified by SEC using a Superose 6.

### Nucleosome core particle (NCP) reconstitution

NCP was reconstituted as previously described (27). Briefly, core histones (His-tagged H2A, H2B, H3 and His-tagged H4) were co-expressed in *E. coli* BL21(DE3) pLysS cells (Novagen). Self-assembled octamer was purified using Talon affinity chromatography under non-denaturing condition in a purification buffer composed of 2M NaCl, 10 mM Tris pH7.0 and 1 mM DTT. After cleaving the His-tag, the octamer was further purified by size exclusion chromatography (SEC) using a Superdex 200 (GE Healthcare). The purified octamer was mixed with a 147-bp double-stranded DNA containing a strong nucleosome-positioning ‘601’ sequence in a 1:1 mole ratio in the purification buffer and the nucleosome was assembled by dialyzing the salt in a step-wise manner.

### *In vitro* methyltransferase assays

Methyltransferase assays were performed as previously described (28,29), and methylated products were either detected by scintillation counting or mass spectrometry. Briefly, reactions were initiated by adding 0.15–3 μM of purified COMPASS with or without CtCfp1 into a reaction mixture containing 3 μM of NCP, 50 mM Tris pH 8.0, 200 mM NaCl, 3 mM dithiothreitol (DTT), 5 mM MgCl_2_, 5% glycerol (assay buffer) and 100 μM *S*-adenosyl-l-methionine (specific activity 800 cpm/pmol). After one-hour incubation, reactions were stopped by spotting the reaction onto Whatman *P*-81 filter papers. Free AdoMet was removed by washing the filter papers as previously described (28,29) and methyltransferase activity was quantified by liquid scintillation counting. H3 peptide substrates corresponding to the first 40 residues of histone H3 either unmodified, mono- or di-methylated on K4 were also used as substrate to examine the kinetics for each methylation steps carry out by COMPASS. Methyltransferase assays were performed in presence of 0.3 μM of purified COMPASS with or without CtCfp1, 0–300 μM peptide substrate, 1 mM *S*-adenosyl-l-methionine in the assay buffer. At each time point, 20 μl of reaction was added into 20 μl of 1% formic acid, flash frozen in liquid nitrogen and then analyzed by ESI-MS to detect the mono-, di-, tri-methylated products. The reaction velocities, determined by using the linear range of product formed versus time, were calculated and plotted *vs* substrate concentrations.

### GST-pull-down assay

GST and GST-fusion proteins were applied onto glutathione-sepharose beads during 1 h at 4°C and washed extensively with GST binding buffer (20 mM Tris, 150 mM sodium chloride, 5 mM β-mercaptoethanol, 0.1% Triton X-100). Beads were then incubated with 10 μg of the purified CtWDR5/MtRbBP5 complex during 2 h at 4°C. Unbound proteins were washed extensively with the binding buffer. Bound proteins were eluted by boiling the beads in SDS loading buffer and analyzed by SDS-PAGE gel.

### Protein crystallization and structure determination

CtCfp1 RID domain (10 mg/ml) was crystallized using the sitting drop vapor diffusion method at room temperature. Diffraction quality crystals were obtained in 0.2 M NH_4_Cl, and 22% (w/v) PEG 3350. Crystals were transferred into the mother liquor supplemented with 20% glycerol, harvested and flash-frozen in liquid nitrogen. A single-wavelength anomalous dispersion (SAD) data set was collected at the 21-ID-D beamline of the Life Science-Collaborative Access Team at the Advanced Photon Source Synchrotron. The structure of CtCfp1 RID was determined by a single wavelength anomalous diffraction at the zinc peak wavelength (Supplementary Table S1). The reflections were processed and scaled using HKL2000 (30) and one zinc atom was identified and refined using the SHELX C/D programs (31). Phases were calculated using SHELX-E and the Arp/Warp program was used to generate the initial model. One chain was traced (95 amino acids out of 186) and used as a search model for molecular replacement using Phaser. The missing residues were modeled in the calculated phases using COOT (32) and the structure was further refined using phenix.refine (33). The final model includes 1 zinc ion, 22 water molecules and CtCfp1 residues 322–404 and 443–508. Owing to the lack of electron density, residues 405–442 were not modeled.

### Inductively coupled plasma mass spectrometry (ICP-MS) metal analysis and isothermal titration calorimetry (ITC)

ICP-MS was performed at the Quantitative Bioelemental Imaging Center, Northwestern University, using a Thermo Fisher X Series II ICP-MS system as previously described (34). Briefly, the MS spectrum of two biological replicates were recorded in triplicate using 100 μM of purified CtCfp1 RID domain or its corresponding buffer (50 mM Tris, pH 8, 200 mM NaCl, 5 mM BME, 2% glycerol). ITC experiments were performed using a VP-ITC calorimeter (MicroCal). A solution of CtCfp1 RID domain (350 μM) was injected into a solution of wild-type MtRbBP5 β-propeller domain (35 μM). Titrations were performed at 20°C in 50 mM Tris pH 8.0, 200 mM NaCl and 2.5 mM β-mercaptoethanol. Titration data were analyzed using the Origin software.

### Cfp1 knockdown

An HEK293 cell line stably expressing a doxycycline (Dox)-inducible shRNA targeting the sequence of 5′ GAGCGATACAAGCGGATC 3′ of Cfp1 was established as follow. HEK293 cells were transfected with a shRNA (V3SH11252-228939455; Dharmacon) cloned in the vector piSMAT-TurboRFP. Following selection using puromycin (2 μg/ml), the sh-resistant WT and mutants of human Cfp1 were generated by site-directed mutagenesis of the shRNA targeting sequence and expressed as Flag-tagged constructs. In the rescue experiments, shRNAs were induced with Dox (0.20 μg/ml) After 48 h, cells were transfected with either Flag-tagged wild-type or mutants of Cfp1. After 24 h, the cells were harvested, washed with PBS and directly lysed with Laemmli buffer. Protein levels were detected by western blot using the indicated antibodies (Cfp1 (ab 19877, Abcam), histone H3 (ab46765, Abcam), H3K4me3 (ab8580, Abcam) and actin (A300-491A, Bethyl lab)).

### Knock out of Cps40 in *Saccharomyces cerevisiae*

SET762 (N-terminally (1-761) truncated Set1) yeast strain was constructed as described before (35). *CPS40* (*Saccharomyces cerevisiae* homologue of Cfp1, ScCfp1) of SET762 strain was knock out by replacing its ORF with *LEU2* auxotrophic marker through PCR-based gene deletion strategy (*SET762 cps40Δ*). Wild-type, truncated, or point-mutant Cps40 with C-terminal 1xFLAG tag was cloned, constructed, and verified in pRS306 plasmid, respectively. Through yeast transformation and homologous recombination of a SET762 cps40Δ strain with PCR-amplified Cps40-1xFLAG-URA3 DNA fragments from each plasmid, SET762 strains harboring wild-type or mutant Cps40-1xFLAG in its original genomic loci were generated. Anti-H3K4me1, H3K4me2 and H3K4me3 antibodies and anti-Set1 antibodies are from Shilatifard laboratory (35). Anti-FLAG antibody is from Millipore Sigma.

### Flag-Cfp1 pull down experiments

Flag-tagged WT and mutants of human Cfp1 were expressed in Cfp1 knockdown cells and harvested for pull-down experiments as previously described (36). Briefly, cells were washed with PBS, and then lysed in the RIPA buffer (50 mM Tris HCl pH 7.4, 400 mM NaCl, 5 mM ethylenediaminetetraacetic acid, 1 mM dithiothreitol, 0.1% NP-40, 0.5% sodium deoxycholate, 25% glycerol and protease inhibitor cocktail). Cell lysis was kept on ice for 30 min before Dounce homogenization. The debris was pelleted by centrifugation at 15 000 rpm, 4°C for 15 min. The supernatant was incubated with pre-equilibrated Anti-FLAG M2 Magnetic Beads (Sigma) during 16 h. Beads were washed four times with buffer containing 300 mM NaCl, 0.1% NP-40 and then boiled in Laemmli sample buffer. Protein levels were probed by western blotting using specific antibodies.

## RESULT AND DISCUSSION

### Cfp1 modestly activates COMPASS methyltransferase activity *in vitro*

**Figure 1.**
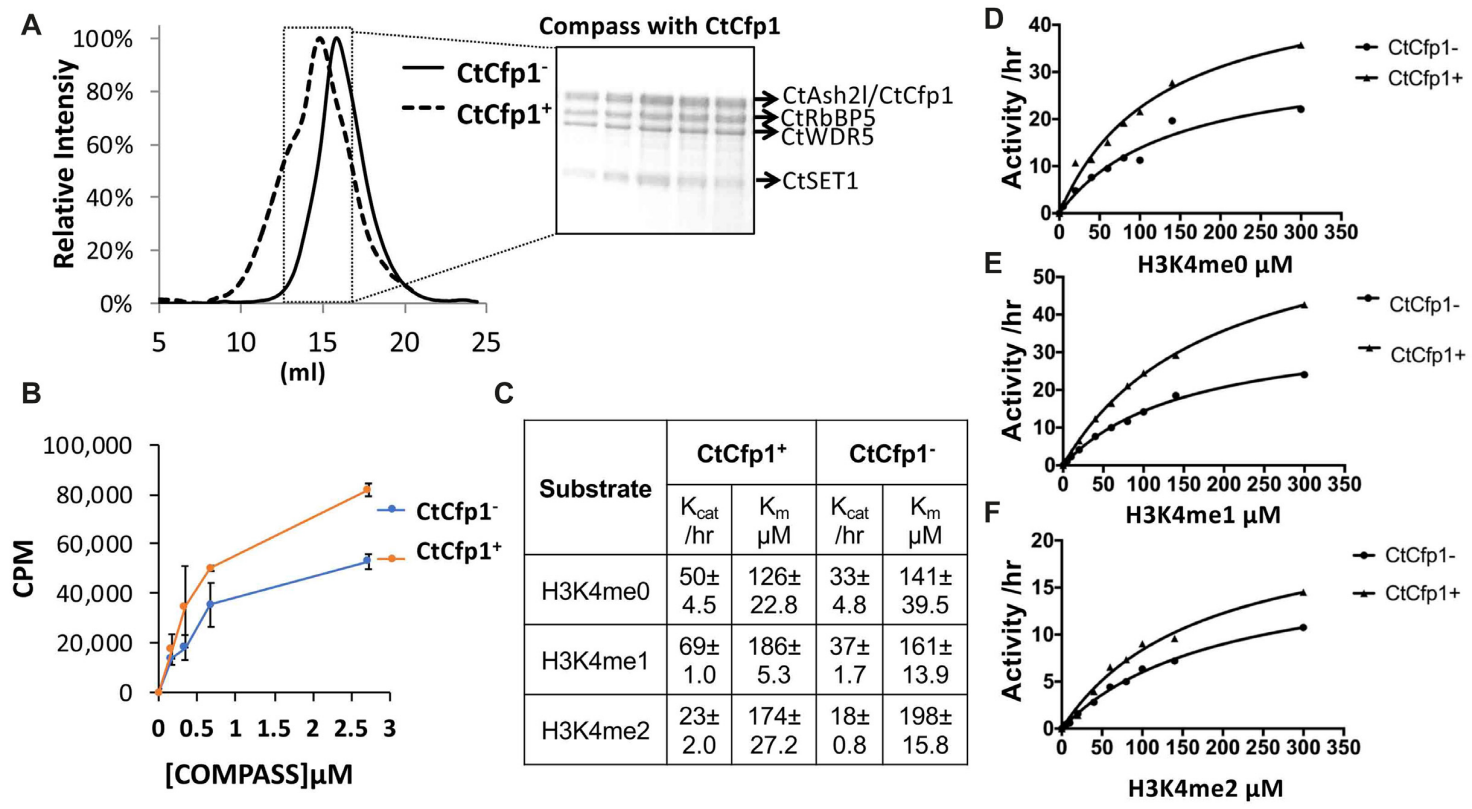
CtCfp1 marginally activates COMPASS methyltransferase activity *in vitro*. (**A**) Reconstitution of COMPASS with CtCfp1. COMPASS was incubated with a 5-molar ratio of CtCfp1 and separated on a superpose 6. The panel shows the chromatogram of COMPASS separated in presence (dotted line) or absence of CtCfp1 (full line). (**B**) CtCfp1 modestly activates the methyltransferase activity of COMPASS on the nucleosome core particle. The error bars indicate the standard deviation of two biological replicates performed in triplicates. (**C**) CtCfp1 modestly activates the methyltransferase activity of COMPASS on peptides. Table showing the kinetic parameters obtained with the michealis-menten plots for H3K4me0 (**D**), H3K4me1 (**E**) and H3K4me2 (**F**) peptides.

Deletion of Cfp1 leads to a sharp loss in H3K4me3 (20) and disrupts programmed double-strand break (DSB) during meiotic recombination in *S. cerevisiae* (16,17). Yet, a recent cryo-EM structure (26) places Cfp1 ∼60 Å (Supplementary Figure S1) away from SET1 catalytic site begging the question; does Cfp1 participates in the positive allosteric regulation of SET1 methyltransferase activity? To address this question, we first sought to homogeneously reconstitute COMPASS in presence or absence of Cfp1. Inclusion of Cfp1 results in a detectable shift in the size of the complex by SEC (Figure 1A) suggesting that Cfp1 stably associates to COMPASS but is dispensable for the assembly of the entire complex. These results are in stark contrast to previous studies showing that omission of RbBP5 leads to a complete disassembly of the complex (26) but can be rationalized by the location of Cfp1 in the cryo-EM structure. RbBP5 contacts each subunit of the complex while Cfp1 is in close proximity of RbBP5 β-propeller domain and a region preceding the catalytic domain of SET1 only (26). Consistent with the long distance separating Cfp1 and SET1, methylation of the nucleosome with COMPASS in presence of Cfp1 only shows a 2-fold increase compared to the complex devoid of the DNA binding protein (Figure 1B). Considering that loss of Cfp1 in yeast leads to a loss of H3K4me3 predominantly, we posited that it may only impact the conversion of H3K4me2 to H3K4me3; a reaction that may form a minute fraction of the product formed in our assays with the nucleosome. To test this hypothesis, we incubated COMPASS, either in presence or absence of Cfp1, with increasing concentration of H3K4me0, H3K4me1 or H3K4me2 peptides and monitored methylation over time by quantitative mass spectrometry. As shown in Figure 1C–E, in absence of Cfp1, COMPASS methylates H3K4me0 and H3K4me1 with similar *k*_cat_ and *K*_m_ values while the turnover number for the H3K4me2 peptide is ∼2-fold slower when compared to the unmethylated substrate (Figure 1C and F). The complex assembled with Cfp1 shows a modest increase in the rates of methylation for H3K4me0 and H3K4me1 peptides with however negligible differences in the Km for these substrates. Similarly, Cfp1 displays negligible effects on the Km of COMPASS for the H3K4me2 substrate. Collectively, our results suggest that COMPASS reconstituted with Cfp1 displays modestly increased *k*_cat_ values for H3K4 peptides with however no preference for a specific methylation state.

### Cfp1 C-terminus associates with the β-propeller of RbBP5

**Figure 2.**
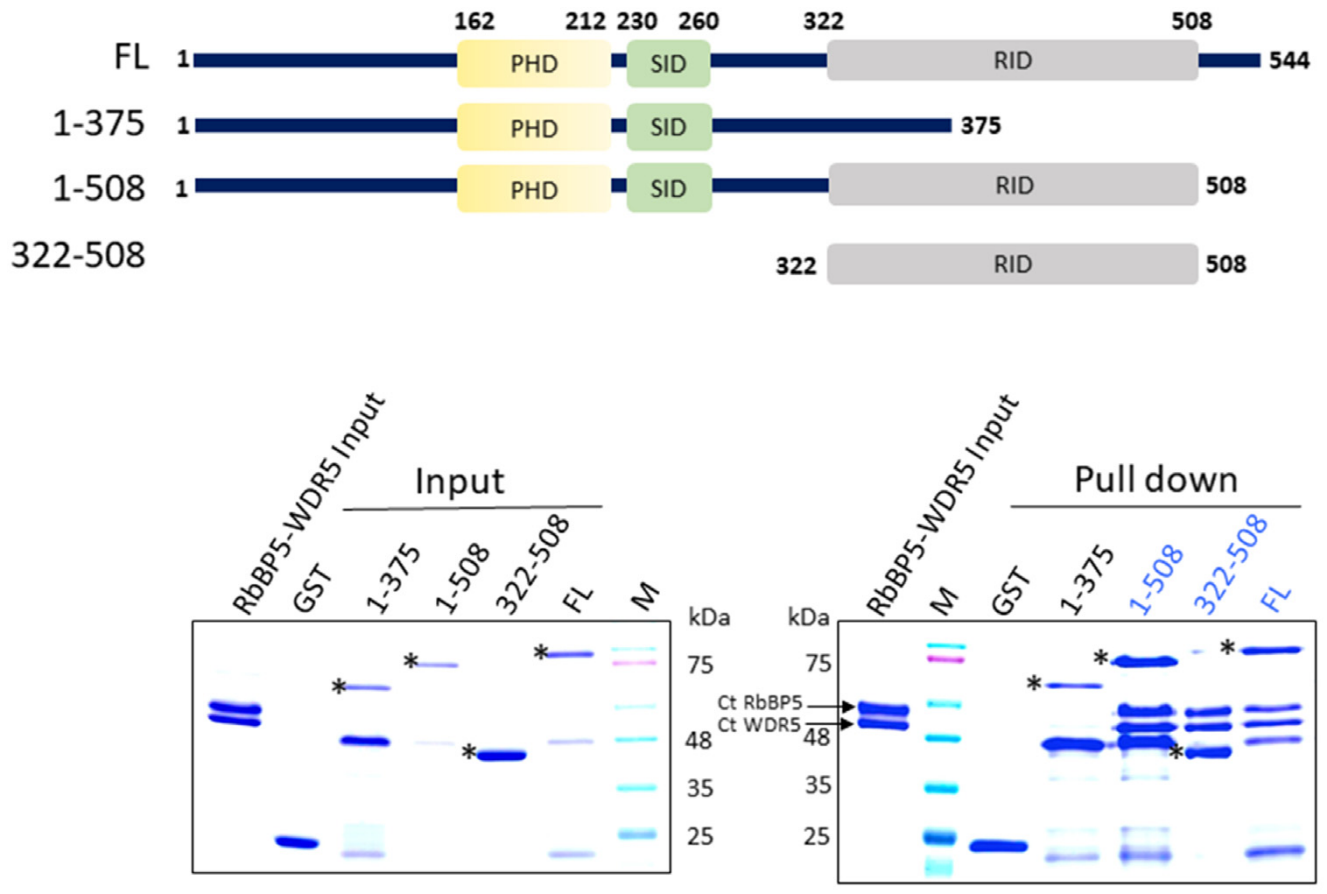
Cfp1 RID domain interacts with RbBP5. (**A**) GST pull down assays showing the interaction between CtCfp1 and RbBP5 (bottom) and schematic representation of *Chaetomium thermophilum* Cfp1 constructs used in the binding studies (top). Proteins were resolved on SDS-PAGE and detected by Coomassie staining. Asterisk (*) indicates the full-length fragments of the constructs tested.

In vertebrates, Cfp1 is composed of a PHD and a CXXC domains, both located on its N-terminus and known to bind H3K4me3 (37) and unmethylated DNA (24), respectively. A central region, referred to as SET Interacting Domain (SID), binds the N-terminus of SET1 (36) while a region located on the C-terminus of the protein is predicted to be composed of a non-canonical PHD domain (Figure 2). To define the biological functions of this domain, we first performed GST-pull down studies with domains spanning different regions of Cfp1. Consistent with the Cryo-EM structure of COMPASS (26) which shows that Cfp1 is found in close proximity of RbBP5 and WDR5 (Supplementary Figure S1), GST-pull down experiments show that Cfp1 directly interacts with the RbBP5/WDR5 heterodimer. With the exception of a construct including its first 508 residues, truncation of Cfp1 C-terminus completely obliterates its binding to the RbBP5/WDR5 heterodimer (Figure 2). Conversely, Cfp1 constructs truncated on its N-terminus maintain its binding activity. Since Cfp1 does not directly interact with WDR5 (Supplementary Figure S2), our results suggest that residues 322–508 are sufficient for RbBP5 binding and will therefore be referred to as RbBP5 interacting domain (RID).

### Cfp1 RID harbors a mono-valent zinc binding site

**Figure 3.**
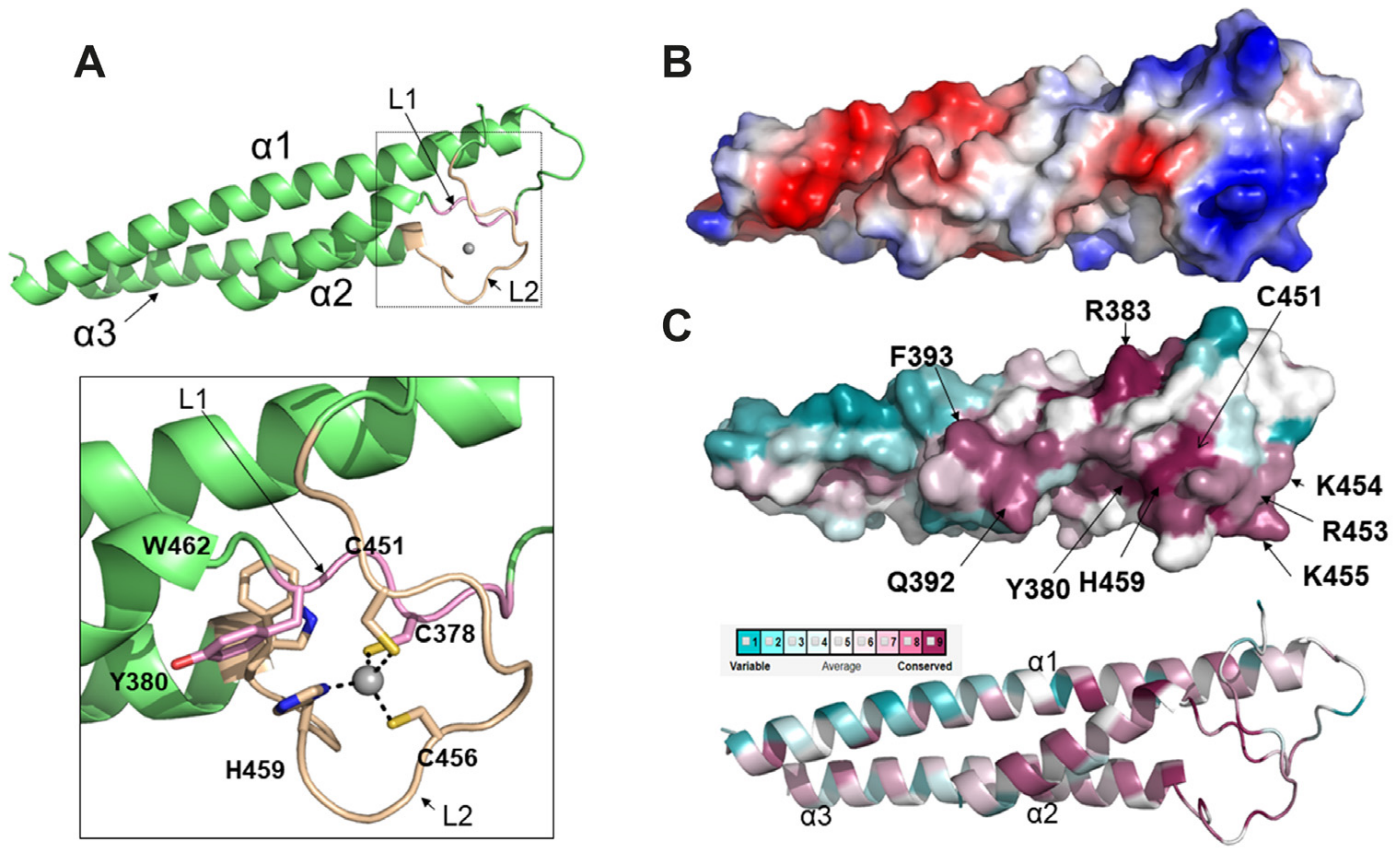
The structure of CtCfp1 RID domain unravels a mono-valent zinc finger. (**A**) CtCfp1 RID domain is formed by a three-helix bundle in which the secondary structure, the zinc binding motifs (I and II) and the zinc atom are rendered in green, light pink, beige and grey, respectively. The inlet shows the residues found in motifs I and II. Zn is coordinated with four ligands. C378 is located in motif I and rendered in light pink. C456, C451 and H459 are found in motif II and are colored in beige. (**B**) Electrostatic potential surface of CtCfp1 RID. Electrostatic surface of Cfp1 RID (blue and red denote positively and negatively charged regions, respectively). (**C**) Conservation of surface residues of Cfp1 RID. Sequence conservation mapped onto Cfp1 are as follows: gradient: dark pink (conserved) to blue (variable).

Cfp1 RID is predicted to contain a single PHD-like domain on its C-terminus. To test this hypothesis, we first measured its metal content using ICP-MS. As shown in Supplementary Table S1, the RID domain contains one zinc atom per RID. Considering that PHD domains are known to bind at least two zinc atoms (38), these observations indicate that the C-terminus of Cfp1 does not fold as a PHD domain. To confirm this, we solved the crystal structure of Cfp1 RID at 2.3 Å (Supplementary Table S2). As defined by the electron density map, Cfp1 RID domain comprises residues 322–508, with one molecule in the asymmetric unit. This region of Cfp1 forms a three α-helix bundle with one Zn^2+^ atom binding site (Figure 3). The metal binding site is formed by two loops (referred to as L1 and L2). L1 is located at the C-terminus of α1 while L2 is located between α2 and α3 (Figure 3A). In L1, one metal coordinating residue, C378, is found in the conserved motif XCGΦ (also referred to as motif I) while in L2, C451, H459, C456 are located in the motif CXXXK/RXXCXXHXXW (also referred to as motif II).

### Cfp1 RID domain zinc finger is evolutionary conserved

Despite its critical role in epigenetic signaling, Cfp1 domain composition has diverged during evolution. *Cheatomium thermophilum* Cfp1 shares 73.8% and 72.0% sequence homology with its *Homo sapiens* and *Sporothrix insectorum* homologs, respectively. The homolog of Cfp1 in *S. cerevisiae* and *Schizosaccharomyces pombe* lacks the CXXC domain and several regions between its SID, PHD and RID domains are poorly conserved (Supplementary Figure S3). However, the surface of Cfp1 RID ZnF domain features a prominent evolutionary conserved and positively charged region (Figure 3B and C). This feature arises from several arginine and lysine side chains including R383, R453, K457 and K455 located in L1 and L2 (Figure 3C). These residues are distributed across the primary sequence of RID but they do not form distinct motifs. In general, the residues forming L1 and L2 are better conserved than the residues found in α-helices indicating that they could mediate an interaction with RbBP5.

### Cfp1 RID znf highlights a novel family of zinc finger

**Figure 4.**
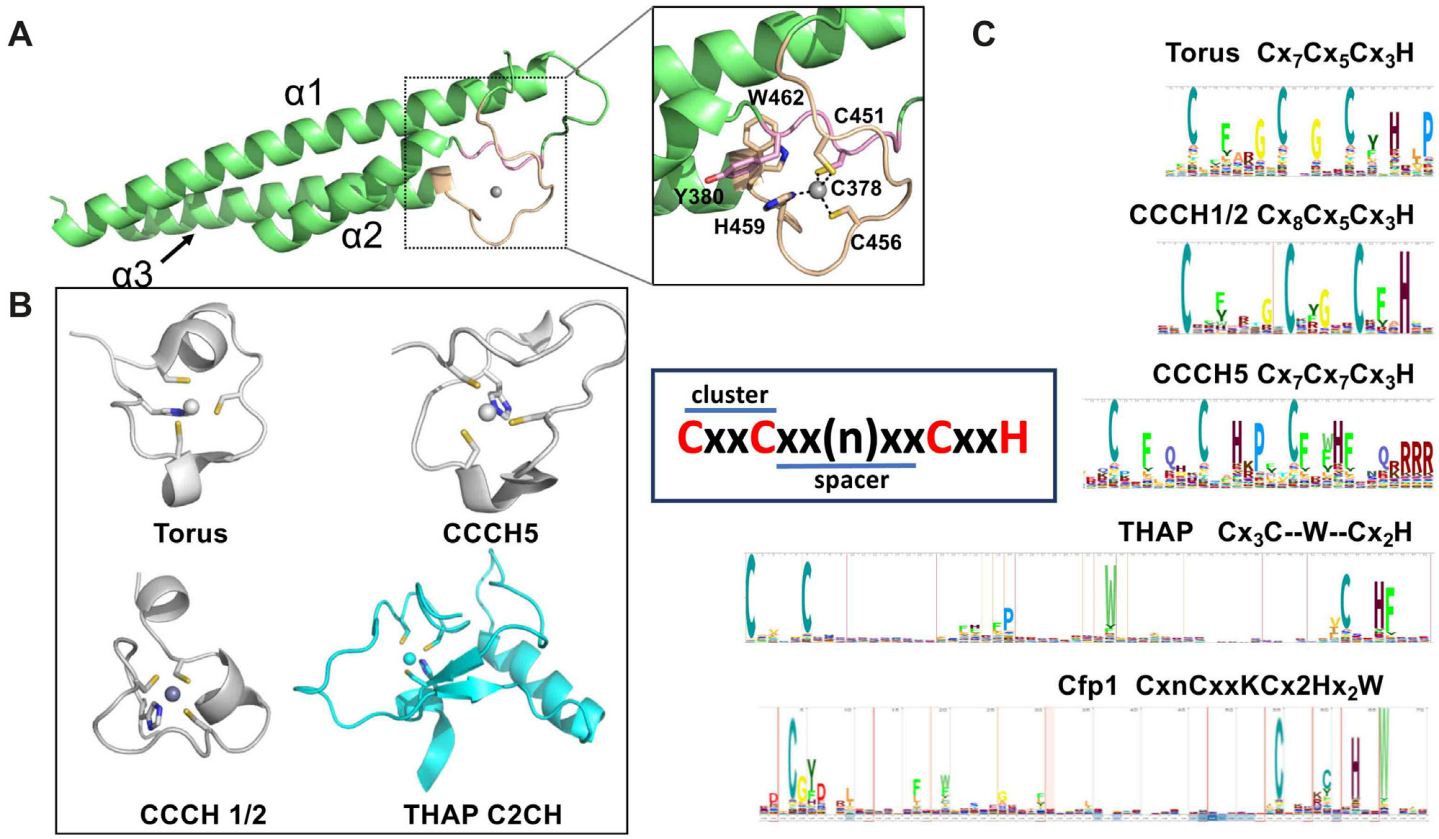
Cfp1 RID ZnF harbors a unique topology. (**A**) Cartoon representation of CtCfp1 RID as shown in Figure 3A. (**B**) Cartoon representation of four types of CxCxCxH zinc finger including Torus (3TP2), CCCH1/2 (4CSA), CCCH5 (5ELH), rendered in gray and THAP (2L1G), highlighted in cyan. Members of the ZC3H and THAP families are colored in grey and cyan, respectively. (**C**) Logo representation highlighting the organization of the metal coordinating residues in Torus, CCCH1/2, CCCH5 and THAP proteins as well as CtCfp1 RID domain. The inlet highlights the cluster and the spacer, which represents a pair of metal coordinating residues and the number of residues separating each cluster, respectively.

Zn fingers (ZnFs) are finger-like protrusions that frequently maintain zinc ions in a tetrahedral geometry typically using a combination of cysteine and histidine residues. While the first ZnF was identified as a DNA-binding domain in transcription factor IIIa (TFIIIA), they are now recognized to play diverse functions and to interact with multiple molecules, including, but not limited to RNA, lipids, and proteins (39,40). Based on the ligand geometry and secondary structure, the HUGO Gene Nomenclature Committee (40–42) classified ZnFs into 30 different families (40–42). Comparative analysis reveals that similar to ZC3H, ZC2HC, ZCCHC, THAP, ZMIZ and PARP-Type zinc fingers, CtCfp1 RID domain employs a trio of cysteine residues and the side chain of a histidine to coordinate the zinc ion (Figure 4A). However, the cysteine residues in the Cfp1 RID domain are contiguous in the primary sequence which is unique to the ZC3H and THAP zinc fingers. Despite these similarities, ZC3H, THAP and Cfp1 RID zinc fingers (Figure 4B) differ in the organization of their ZnF. Logo analysis (Figure 4C) shows that in ZC3H, the first pair (also referred to as cluster) of cysteines is separated (also referred to as spacer) from the other metal-coordinating residues by ∼5 amino acids with a similar separation in each cluster. Analogous to ZC3H, residues forming each pair of metal-coordinating residues of the THAP ZnF is separated by the same number of residues with however a spacer exceeding 50 amino acids. However, in stark contrast to ZC3H and THAP, the residues forming the first cluster in CtCfp1 RID ZnF are separated by ∼80 residues; a distance that exceeds all the other zinc binding domain reported to date. Therefore, these observations suggest that the ZnF of Cfp1 RID is a novel type of ZnF.

### Cfp1 RID ZnF is required for its association to COMPASS

**Figure 5.**
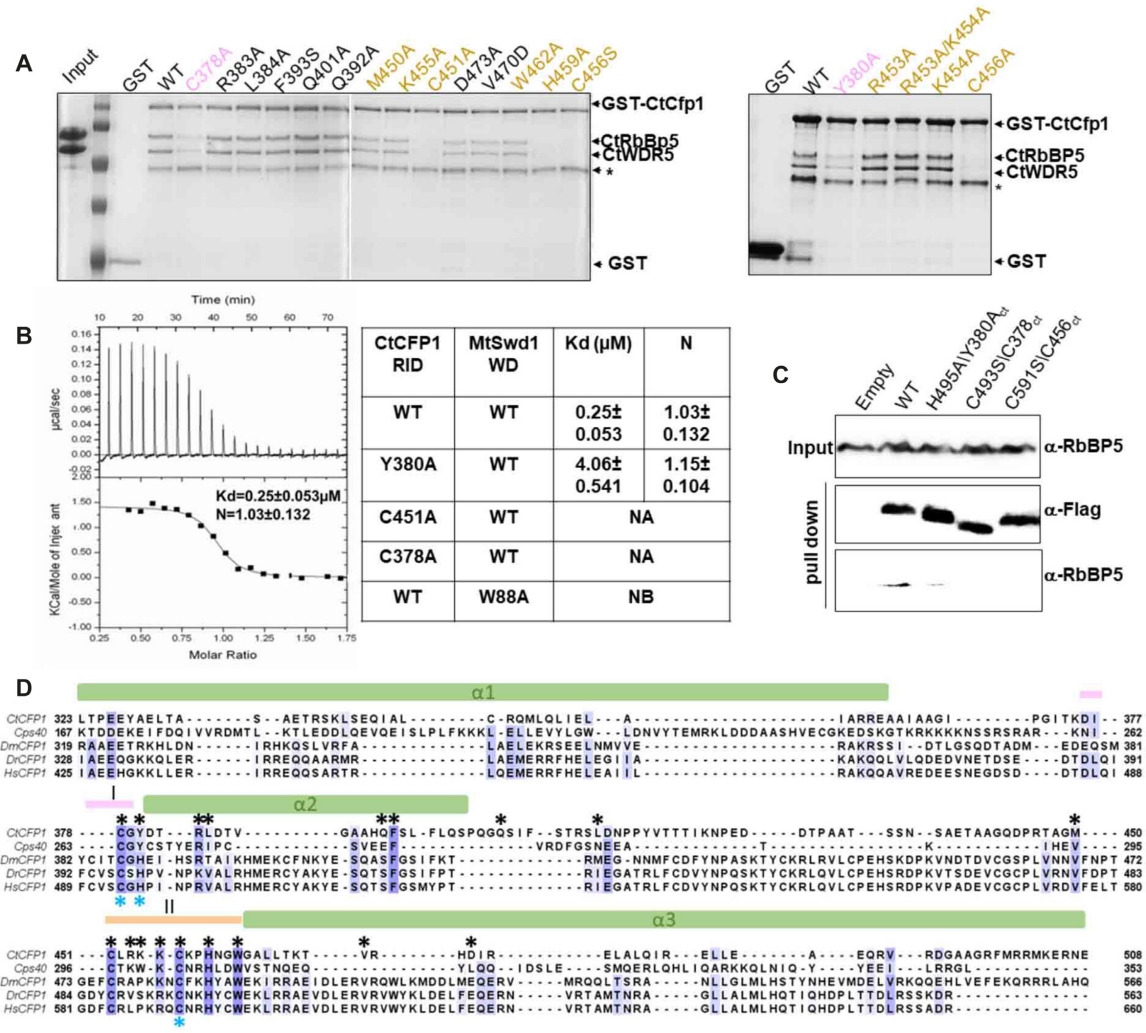
Key residues forming Motif I and II of CtCfp1 RID are important for RbBP5 binding. (**A**) RbBP5/WDR5 complex interacts with Cfp1 RID ZnF. GST-tagged Cfp1 proteins (wild-type or mutants) were incubated with the RbBP5/WDR5 complex. Bound proteins were resolved on a 15% SDS-PAGE and Coomassie-stained. Star (*) indicates a degradation product of GST-tagged Cfp1. The residues located on motif I and Motif II are colored in pink and beige, respectively. (**B**) Isothermal titration calorimetry curve of Cfp1 RID titrated into RbBP5 β-propeller. (C) ITC values for Cfp1 RID wild-type and mutants. Titrations were performed in duplicate. S.D. represents the standard deviation between the two experiments. N.A.: Experiments could not be performed as the mutant aggregated during ITC. N.B.: No binding could be detected. (**C**) Replacement of metal coordinating residues lowers the interaction between Cfp1 and RbBP5. Immunoprecipitation of ectopically expressed FLAG-tagged constructs of Cfp1 wild-type and motifs I and II mutants. Their corresponding residues in *Chaetomium thermophilum (Ct)* are labeled. RbBP5 and FLAG-tagged Cfp1 were detected with indicated antibodies. (**D**) Cfp1 RID ZnF is evolutionary conserved. A protein sequence alignment of Cfp1 RID domain from *Chaetomium thermophilum* (Ct) with the corresponding RID domains of *Saccharomyces cerevisiae* (Cps40), *Drosophila melanogaster* (Dm), *Danio rerio* (Dr), *Homo sapiens* (Hs). RID α-helices are depicted as cylinders and the motifs I/II are highlighted as lines with colors corresponding to the Figure 3. Evolutionary conserved residues are rendered with Blosum62 color scheme. Residues matching the consensus sequence residue at that position are colored in dark blue. Based on the conservation score calculated by Bloosum62 matrix, other residues are coloured in different shades of blue. The black and blue * indicates the residues mutated on CtCfp1 (A) and HsCfp1 (C) respectively.

Owing to the low resolution of the Cryo-EM structure of COMPASS in the region corresponding to the ZnF of Cfp1, the zinc finger had not been modeled in the initial maps. Cfp1 RID domain and its ZnF was aligned with Cps40 fitted in the cryo-EM maps with a good correlation, especially for the two long intertwined helices with a rmsd at ∼1.09Å (Supplementary Figure S4A). Consistent with the analysis of the evolutionary conserved regions on Cfp1 RID domain, L1 and L2 are found in close proximity of Cps50 (yeast RbBP5) in the cryo-EM structure of COMPASS (Supplementary Figure S4). Surprisingly, mutation of several residues (Arg383, Leu384, Phe393, Gln392, Gln401, Met450, Lys455, Asp473, Val470 and Trp462) (Figure 5A) fail to impair Cfp1 binding to RbBP5. However, mutation of the residues forming both motifs and Tyr380 severely impede the binding of RbBP5 to Cfp1 (Figure 5A). This is illustrated by a ∼20-fold loss in binding for the Tyr380 substituted by an alanine when compared to wild-type Cfp1 (Figure 5B). Correspondingly, these mutants fail to associate with RbBP5 in HEK293 cells (Figure 5C) suggesting that Cfp1 RID ZnF is important for binding to RbBP5.

### The ZnF of the RID domain is required for H3K4 tri-methylation in mammalian cells and yeast

**Figure 6.**
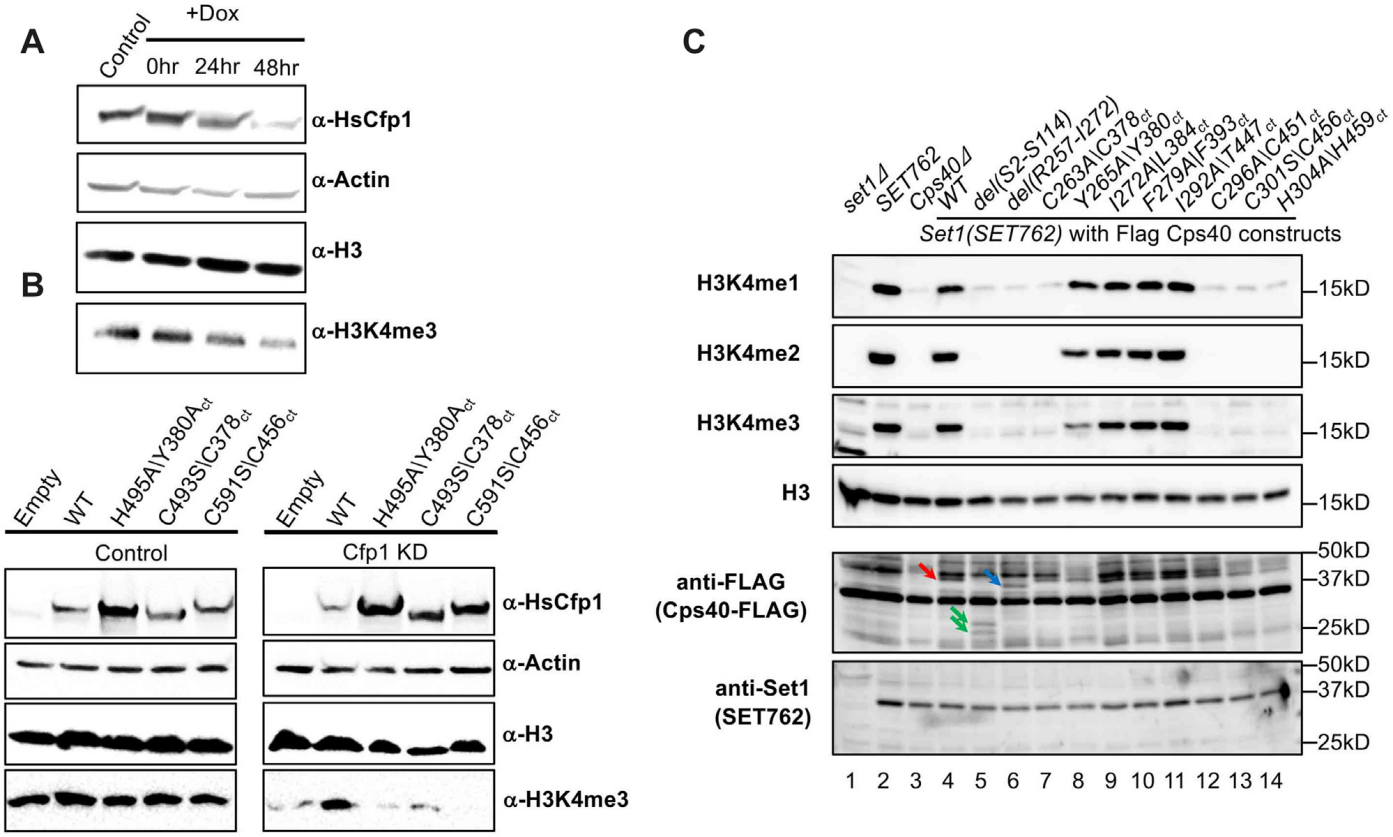
Retention of RbBP5-binding activity of Cfp1 is required for appropriate H3K4 methylation. (**A**) Western blot analysis was performed on cellular extracts isolated following incubation of HEK393 cells with dox-inducible sh-RNA targeting Cfp1. Cfp1, actin, histone H3 and H3K4me3 levels were detected with indicated antibodies. (**B**) Immunoblotting of cellular extracts following the transfection of dox treated HEK293 cells with sh-resistant constructs of Cfp1 wild-type or mutant. Protein levels were detected as in panel 1A. The corresponding residues in *Chaetomium thermophilum (Ct)* are also indicated. (**C**) Immunoblotting of cellular extracts from *Saccharomyces cerevisiae* strain in which Cps40 was either deleted or rescued with flag tagged constructs corresponding to full length Cfp1 wild type or mutants. The corresponding residues in *Chaetomium thermophilum (Ct)* are also indicted. Flag-Cfp1, SET1, histone H3 and H3K4 methylation levels were detected with indicated antibodies. Arrows indicate full-length Cfp1 (red), and Cps40 constructs containing an internal deletion (blue) or a truncation of its N-terminus (green), respectively. The experiments were performed using in a SET762 background.

Knockdown of Cfp1 in mouse oocytes (13), T-cells (43) GM-CSF derived macrophages (44) and its knock-out in budding yeast (22,35) results in a decrease of H3K4me3. To test the impact of mutations impairing Cfp1/RbBP5 complex formation on histone H3K4 methylation, we transfected FLAG-tagged constructs corresponding to Cfp1 WT (Cfp1^WT^), H495A (Cfp1^H495A^), C493S (Cfp1^C493S^) or C591S (Cfp1^C591S^) in HEK293 cells stably expressing a doxycycline (Dox)-inducible small hairpin shRNA directed against Cfp1. Treatment of the cells with Dox resulted in a loss of Cfp1 and reduction of H3K4me3 (Figure 6A) while their transfection with sh-resistant FLAG-Cfp1^WT^ restored the PTM to that of the wild type level (Figure 6B). Consistent with our binding assays, transfection with FLAG-Cfp1^H495A^, FLAG-Cfp1^C493S^ or FLAG-Cfp1^C591S^ constructs failed to rescue the loss of H3K4me3 (Figure 6B). To gain a deeper understanding of Cfp1/RbBP5 interaction, we generated yeast strains either lacking Cps40 (*Cps40Δ*) or Cps40 deleted strain rescued with Cps40 wild-type or mutants of its RID ZnF (Figure 6C). Consistent with previous studies (20,45), deletion of Cps40 resulted in an apparent complete loss of H3K4 methylation (Figure 6C). Truncation of motif I (a.a 257–272) or mutation of the Zn-binding residues C263, C296, C301, or H304, resulted in the same phenotype as the deletion of Cps40. Similarly, substitution of Y265 for an alanine reduced H3K4me3 significantly. Consistent with GST pull down assays, rescue experiments with other mutants targeting the evolutionary conserved residues such as I272, F279 and I292 did not impair H3K4 methylation. Interestingly, deletion of the N-terminus (a.a 2-114), which include the first PHD and the SID, completely blunted H3K4 methylation, suggesting the Cfp1 multivalent interactions within COMPASS and H3K4me3 binding activity contribute to epigenetic signaling. This model is further supported by methyltransferase assays showing that Cfp1 RID is insufficient to stimulate COMPASS methyltransferase activity (Supplementary Figure S5).

### The contribution of Cfp1 to the activity of SET1 methyltransferases

Our biochemical studies show an apparent disconnect between the contribution of Cfp1 to SET1 methyltransferase activity *in vivo* and *in vitro*. In yeast, loss of Cfp1 results in a sharp decrease in H3K4 methylation yet its incorporation in the purified complex leads to only a modest increase in the stimulation of its methyltransferase activity *in vitro*. Consistent with previous single time point experiments (46), these observations are in stark contrast to other subunits of the complex. For example, akin to their impact on H3K4 methylation *in vivo* (5), Ash2L and RbBP5 stimulate the methyltransferase activity of KMT2 enzymes between 200–600 folds approximately (4). However, the modest stimulation by Cfp1 is similar to Dpy-30. *In vitro*, Dpy-30 increases KMT2 enzymes activity by only 2-fold (47) but its loss leads to a sharp decrease in global H3K4me3 in *S. cerevisiae*. Several models have been proposed for the regulatory mechanism of Cfp1. First, Cfp1 activates SET1 for H2Bub-dependent H3K4 trimethylation through an interaction with a region preceding the catalytic domain of SET1 (22). However, this model is not supported by recent chromatin immunoprecipitation (ChIP) experiments in *S. cerevisiae* which show that H2Bub levels are relatively consistent throughout transcribed regions (48,49) yet the distribution pattern of H3K4me3 shows an enrichment near the promoter region of transcribed genes with a steady decline toward their 3′ends (50–52). In the second model, binding of Cfp1 to SET1 stabilizes the enzyme *in vivo*. Unfortunately, this model does not explain the persistence of H3K4me2 and H3K4me1 in yeast following the loss of Cps40 (20). In an alternative model, H3K4 methylation patterns reflect the transcriptional activity at a specific promoter (50). Consistent with the ability of COMPASS to bind the phosphorylated C-terminal heptapeptide repeat (CTD) of RNA polymerase II (9,53), the third model suggests that H3K4me2 and H3K4me3 levels reflects the polymerase turnover at a given promoter (50). This model was recently supported by structural and ChIP-seq studies highlighting the heterogeneity in the distribution of H3K4me3 following the loss of Dpy-30 (47). Our observations that the absence of Cfp1 has a moderate effect on each methylation step *in vitro* but still blunt H3K4me3 in HEK293 cells and in budding yeast shows that the cumulative drop of 3.6-fold for the k_cat_ in absence of Cfp1 *in vitro* is sufficient to disrupt epigenetic signaling *in vivo*. This correlates well with the ∼80% loss of H3K4me3 yeast following the deletion of Cfp1 in yeast (20). Our observations are also consistent with ChIP-seq profiling of H3K4me2 following the deletion of yeast Cfp1 (50). These studies show a displacement of H3K4me2 peaks toward the TSS which reflect the reduced rates of H3K4 methylation by a COMPASS lacking Cfp1 at RNA polymerase bound promoters (35). However, our data does not rule out that ubiquitinated nucleosome is a better substrate for COMPASS *in vitro*. In that scenario, the absence of Cfp1 would negatively impact all the methylation steps presumably affecting H3K4 methylation levels even more in chromatin regions marked by H2Bub. Collectively, our results suggest that even a modest loss of stimulation of KMT2 enzymatic activity *in vitro* could lead to a significant loss of H3K4 tri-methylation *in vivo*.

## CONCLUSIONS

Cfp1 is a multifunctional protein harboring several functional domains that contribute to H3K4 methylation. First, the PHD domain of Cfp1 binds H3K4me3 and contribute to the appropriate enrichment of Cfp1 at non-methylated CpG islands with elevated H3K4me3 (11). In metazoans, its CXXC domain directly binds unmethylated CpG islands (18,24) while its SID region stabilizes Cfp1 integration into COMPASS formed of SET1A/B enzymes (22). In this study, we highlighted a fourth domain and a non-canonical form of ZnF that is responsible for the integration of Cfp1 into COMPASS in binding to RbBP5. Considering that gene duplications of the SET1A gene is found in almost half of breast cancer (54,55) and the essential role of Cfp1 in controlling the activity of SET1A/B, our studies may have unraveled a novel pocket for structure-activity relationship studies and the development of small molecules able to disrupt this region of COMPASS.

## Supplementary Material

gkz1037_Supplemental_File
