# Supplementary material for: A non-canonical monovalent zinc finger stabilizes the integration of Cfp1 into the H3K4 methyltransferase complex COMPASS
Source: Nucleic Acids Res. 2019 Nov 14;48(1):421–31. doi: 10.1093/nar/gkz1037 (PMC7145517; doi:10.1093/nar/gkz1037)
Supplement: gkz1037_Supplemental_File [file gkz1037_supplemental_file.pdf]

Supplemental information for the manuscript:

A non-canonical monovalent zinc finger stabilizes the integration of Cfp1 into SET1 complexes within COMPASS.

Yidai Yang[1], Monika Joshi[1], Yoh-hei Takahashi[2], Zhibin Ning[1], Qianhui Qu[3], Joseph S. Brunzelle[4], Giorgios Skiniotis[3], Daniel Figeys[1], Ali Shilatifard[2] and Jean-François Couture[1]

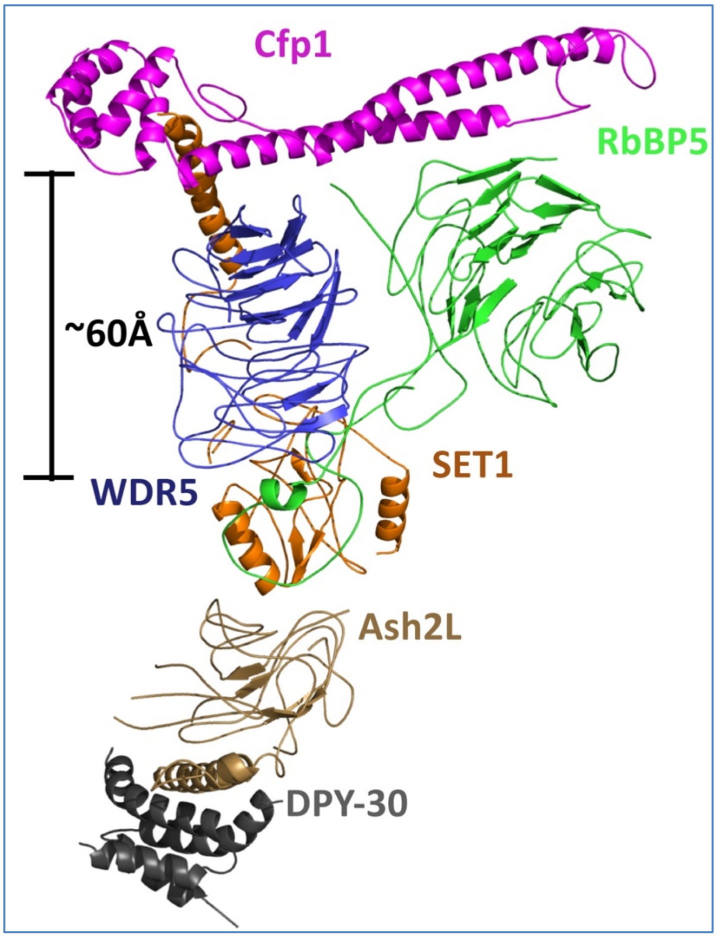

Figure S1. The Cryo-EM model of COMPASS. Colors are rendered related subunits: Cfp1 (pink), RbBp5 (green), WDR5 (blue), SET (orange), Ash2L (sand), DPY-30 (grey). Cfp1 is about 60 Å away from SET domain.

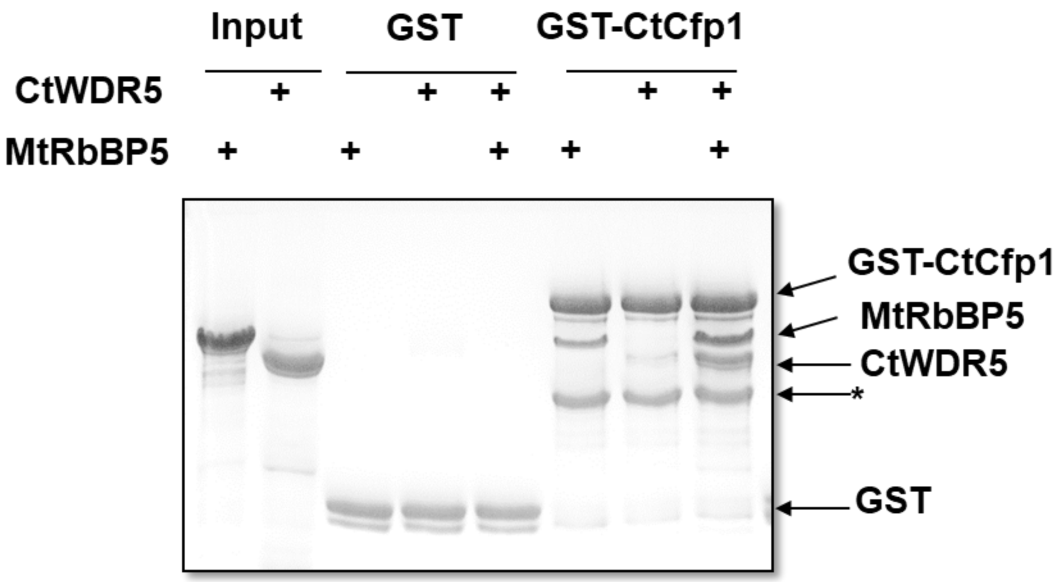

Figure S2. Cfp1 tightly interacts with RbBP5 but not WDR5. GST-tagged Cfp1 were incubated with RbBP5, WDR5 or RbBP5/WDR5 complex. Bound proteins were separate on a 15% SDS-PAGE and detected by Coomassie staining. Star (*) indicates a degradation product of GST-tagged CtCfp1.

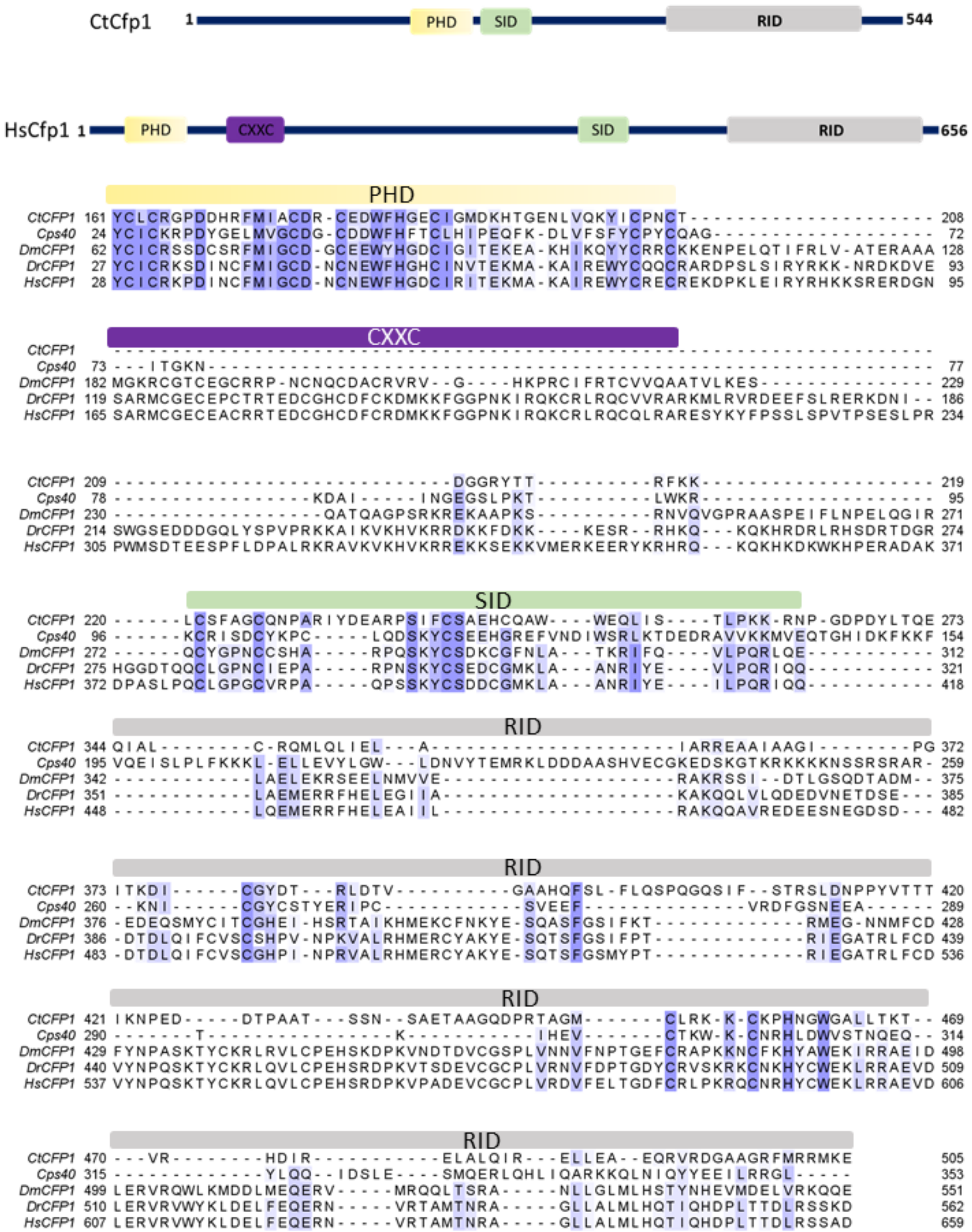
CtCfp1
1
PHD
SID
RID
544
HsCfp1
1
PHD
CXXC
SID
RID
656
PHD
CXXC
SID
RID
CtCFP1
Cps40
DmCFP1
DrCFP1
HsCFP1

Figure S3. Sequence alignment of Cfp1. Top: domain structure of Cfp1. Bottom: A protein sequence alignment of full-length Cfp1 from *Chaetomium thermophilum (Ct), Saccharomyces cerevisiae (Cps40), Danio rerio (Dr), Drosophila melanogaster (Dm), and Homo sapiens (Hs).* The PHD (Yellow), CxxC (Purple), SID (green), and RID (grey) domains are shown on the top of the corresponding sequence. Evolutionary conserved residues are highlighted with Blosum62 color scheme. Residues matching the consensus sequence at that position are colored in dark blue while less conserved residues are rendered using different shades of blue. CtCfp1 residues 1-160, 274-343, and506-544 were omitted from the alignment.

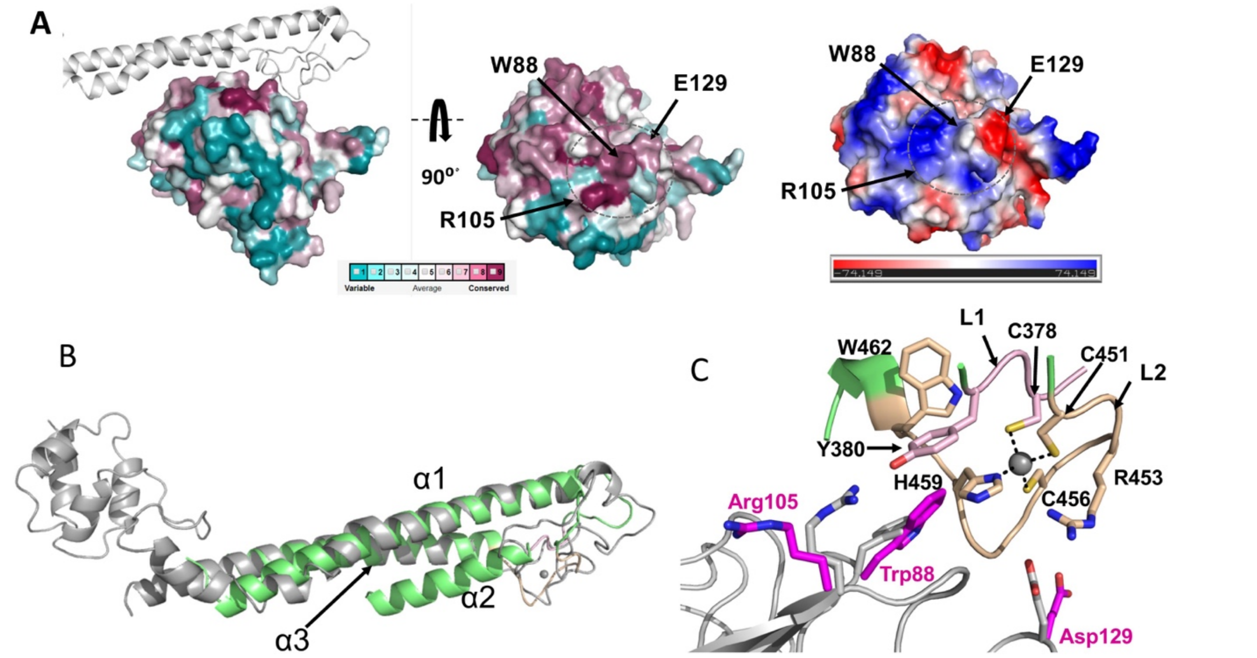

Figure S4. Interaction between Cfp1 RID and RbBP5 β-propeller. A) ZnF binding site on RbBP5 is conserved. Crystal structure of MtRbBP5 was docked into the cryo-EM structure of COMPASS. Cfp1 is rendered in grey and conservation of RbBP5 β-propeller surface residues are colored as in Figure 2. The middle panel shows an orthogonal view of RbBP5 β-propeller. Electrostatic potential surface of RbBP5 β-propeller is shown in the right panel. Electrostatic potentials are contoured from +10kbTe^{-1} (blue) to -10 kbTe^{-1} (red). B) Overlay of Cfp1 crystal structure (green) on top of the Cps40 cryo-EM structure (grey). Helix α1 and α3 of Cfp1 and Cps40 aligns with r.m.s.d. of ~1A. C) A zoomed view of Cfp1 RID ZnF. Cfp1 RID ZnF is colored as in Figure 2. The residues found to impact the interaction between RbBP5 and Cfp1 are highlighted. The residues on Cfp1 are labeled as one-letter code, while residues on MtRbBP5 in the cryo-EM structure are labeled as a 3-letter code. The residues from MtRbBP5 crystal structure are were overlaid on the top of Cps50 (yeast homolog in RbBP5, rendered in grey) from the COMPASS cryo-EM structure and rendered in magenta.

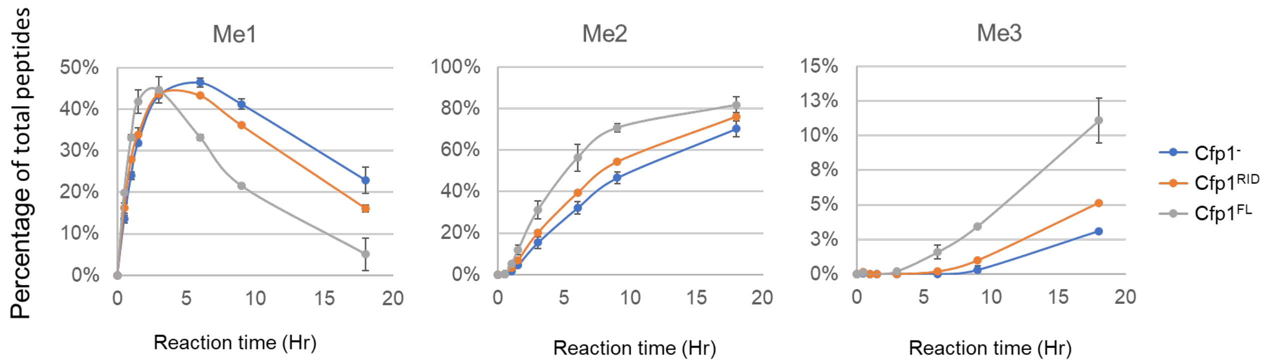

Figure S5. RID of Cfp1 alone is not sufficient to fully activate COMPASS. Methyltransferase assays were performed in presence of 0.3 μM of purified COMPASS with or without CtCfp1, H3 peptide substrates corresponding to the first 40 residues of histone H3 and S-adenosyl-L-methionine in the assay buffer. The proportion of H3K4me1 (left), H3K4me2 (middle) and H3K4me3 (right) peptides were reported as a percentage of the total amount of peptide used in the assay and reported on the Y-axis. Error bars indicate the s.d. of two independent experiments performed in triplicate.

TABLES

**Supplementary Table S1 : ICP-MS analysis of purified Cfp1 RID domain concentrated at 100μM.**	
**Sample**	***Zn concentration (μM)***
protein	119.41 ± 5.31
Buffer	0.04 ± 0.01

**Supplementary table S2: Data collection and refinement statistics for the CtCfp1 RID domain**	
PDB Accession number	
**Data collection**	
Space group	I222
Cell dimensions	
a, b, c (Å)	67.3, 71.6, 78.1
Resolution (Å)	51.98 – 2.31 (2.44 – 2.31)
R_{meas}	0.043 (0.377)
$I / \sigma I$	15.0 (2.7)
Completeness (%)	99.2 (96.7)
Redundancy	13.3 (13.0)
CC1/2	0.998
**Refinement**	

Resolution (Å)		49.0 – 2.3
No. reflections		8459
R_{work} / R_{free}		23.5/27.7
No. Atoms		
	CtCfp1	1075
	Zn^{2+}	1
	Water	22
B-factors (Å^2)		
	Protein	52.2
	Zn^{2+}	75.6
	Water	47.9
R.m.s. deviations		
	Bond lengths (Å)	0.008
	Bond angles (°)	0.904
Molprobity scores		1.24
Ramachandran favored (%)		97.8
Ramachandran allowed (%)		1.2

! Highest resolution shell is shown in parentheses.
